# A cross-cultural comparison of folk plant uses among Albanians, Bosniaks, Gorani and Turks living in south Kosovo

**DOI:** 10.1186/s13002-015-0023-5

**Published:** 2015-05-12

**Authors:** Behxhet Mustafa, Avni Hajdari, Andrea Pieroni, Bledar Pulaj, Xhemajli Koro, Cassandra L Quave

**Affiliations:** Institute of Biological and Environmental Research, University of Prishtina “Hasan Prishtina”, Mother Teresa, 1000 Prishtinë, Kosovo; University of Gastronomic Sciences, Piazza Vittorio Emanuele 9, I-12060 Pollenzo, Italy; Center for the Study of Human Health, Emory University, 550 Asbury Circle, Candler Library 107E, Atlanta, GA 30322 USA; Department of Dermatology, Emory University School of Medicine, 1518 Clifton Rd NE, CNR Bldg. 5000, Atlanta, GA 30322 USA

**Keywords:** Ethnobotany, Sharr Mountains, Folk medicine, Kosovo, Medicinal plants, Wild food plants

## Abstract

**Background:**

Kosovo represents a unique hotspot of biological and cultural diversity in Europe, which allows for interesting cross-cultural ethnobotanical studies. The aims of this study were twofold: 1) to document the state of traditional knowledge related to local (esp. wild) plant uses for food, medicine, and handicrafts in south Kosovo; and 2) to examine how communities of different ethnic groups in the region (Albanians, Bosniaks/Gorani, and Turks) relate to and value wild botanical taxa in their ecosystem.

**Methods:**

Field research was conducted in 10 villages belonging to the Prizren municipality and 4 villages belonging to the Dragash municipality, located in the Sharr Mountains in the southern part of Kosovo. Snowball sampling techniques were used to recruit 139 elderly informants (61 Albanians, 32 Bosniaks/Gorani and 46 Turks), for participation in semi-structured interviews regarding the use of the local flora for medicinal, food, and handicraft purposes.

**Results:**

Overall, we recorded the local uses of 114 species were used for medicinal purposes, 29 for food (wild food plants), and 20 in handicraft activities. The most important species used for medicinal purposes were *Achillea millefolium* L., *Sambucus nigra* L., *Urtica dioica* L., *Tilia platyphyllos* Scop. *Hypericum perforatum* L., *Chamomilla recutita* (L.) Rauschert, *Thymus serpyllum* L. and *Vaccinium myrtillus* L. *Chamomilla recutita* was the most highly valued of these species across the populations surveyed. Out of 114 taxa used for medicinal purposes, only 44 species are also included in the European Pharmacopoeia. The predominantly quoted botanical families were Rosaceae, Asteraceae, and Lamiaceae. Comparison of the data recorded among the Albanian, Bosniak/Gorani, and Turkish communities indicated a less *herbophilic* attitude of the Albanian populations, while most quoted taxa were quoted by all three communities, thus suggesting a hybrid character of the Kosovar plant knowledge.

**Conclusion:**

Cross-cultural ethnobiological studies are crucial in the Balkans not only for proposing ways of using plant natural resources, which could be exploited in sustainable local development projects (e.g. focusing on eco-tourism and small-scale trade of medicinal herbs, food niche and handicrafts products), but also for fostering collaboration and reconciliation among diverse ethnic and religious communities.

## Introduction

Over the last decade, the Western Balkans have become the arena of a remarkable number of ethnobiological field studies, which have focused on territories of Bosnia and Herzegovina [1-7], Serbia [8-12], Montenegro [13], Albania [14-19], Macedonia [20-24], and Kosovo [25,26]. Moreover, a few of these studies addressed cross-cultural comparisons in an attempt to try to understand cultural concepts underpinning perceptions and uses of specific plants, especially among Albanian vs. Slavic populations [10,15,21]. Much of this focus on Balkan ethnobotany is linked to the long and ongoing history of gathering and trading local wild medicinal plants from this territory into Western European markets. It is also supported by the growing appreciation of ethnobotanical bio-cultural heritage as a starting point for fostering a peaceful and sustainable development in the area.

As part of our ongoing long-term project of documenting the ethnobotanical knowledge of diverse multi-cultural and religious areas in the Balkans, here we focused our attention on the Prizren and Dragash municipalities (South Kosovo), where traditionally diverse ethnic groups (Albanians, Turks, Bosniaks, Serbians, Gorani, Roma/Gypsies, Egyptians and Ashkali) have lived in close contact for many centuries. Previous ethnobotanical and ethnolinguistic studies conducted in Kosovo have demonstrated that medicinal plants still play a crucial role in the sphere of human health, especially in isolated rural areas [25-27]. Oftentimes, these mountainous communities have limited access to Western biomedical facilities, and they rely heavily on traditional ecological knowledge (TEK) to meet their dietary and medical needs. It is for this reason that we project that investigation of Kosovo’s diverse ethnobotanical heritage will have a tremendous impact on rural development projects aimed at improving the holistic and long-term well-being of the local populations via sustainable use of local natural resources and integration of emic concepts of health and dietary care into development plans.

The aims of this study were twofold: 1) to document the state of traditional knowledge related to local (esp. wild) plant uses for food, medicine, and handicrafts in southwest Kosovo; and 2) to examine how communities of different ethnic groups in the region (Albanians, Bosniaks/Gorani, and Turks) relate to and value wild botanical taxa in their ecosystem.

## Methods

### The study area

In this study, we investigated traditional ecological knowledge (TEK) concerning the use of local plants in villages situated in the territory of Prizren, which lies in the southwestern part of the Sharr Mountains (in Albanian known as *Malet e Sharrit*; in Serbo-Croatian as *Šar Planina*). The Sharr Mountains lie in the Republic of Macedonia and Kosovo and have a total area of 1,600 km^2^. The Republic of Macedonia is home to 51% (827 km^2^) of this mountain range, while the Republic of Kosovo is home to the rest (780 km^2^) [28]. The Sharr Mountains provide an interesting site of plant life richness and diversity, with an estimated 2,000 vascular plant species. Indeed, a special characteristic of the Sharr Mountains is the presence of endemic, relict, and rare species and plant communities [29]. The most representative vegetation includes black alder communities *(Alnetum glutinosae*), which is widespread along the streams and rivers, oriental hornbeam forest (*Carpinetum orientalis scardicu*), hop hornbeam mixed with oriental hornbeam forest (*Ostryo-Carpinion orientalis*), thermophilous oak forests (*Quercetum frainetto-cerris scardicum,* and *Quercetum pubenscens*, *Quercetum montanum*, *Quercetum trojanae dukagjini*)*,* beech forests (*Fagetum montanum*), and pine forests (*Pinetum heldreichii*, *Pinetum peucis, Pinetum mughi typicum*) [30].

**Figure 1:**
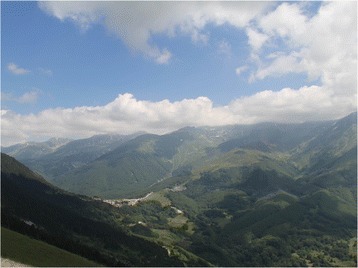
Landscape of the Sharr National Park.

In recognition of the rich levels of biodiversity in this region, in 1986 the Kosovo Assembly (former Autonomous Province of Kosovo within the Socialist Federal Republic of Yugoslav) declared that a part of the Sharr Mountains would be a National Park with the size of around 30,000 hectares. In 2012, the borders of the National Park were expanded and at the same time the massif of Koritnik was included, increasing the park’s territory by around 23,469 hectares. Now recognized as the Sharr National Park (Figure 1), it occupies 53,469 hectares, and includes the territories of five municipalities: Kaçanik, Shtërpcë, Suharekë, Prizren and Dragash [31].

Over the past two millennia, this region has been continuously occupied and was part of three great empires (Roman, Byzantine and Ottoman). In the intervals between the decline of one emperor and empowerment of another, Kosovo was occupied mainly by Bulgarians and Serbs. According to Schmitt [32], when the Romans arrived in the Kosovar territory, they were faced with various Illyrian tribes. In late antiquity, the Dardanians became mainly Christian (Catholic); Byzantine and Slavic invasions led to the Catholicism of a significant proportion of indigenous Albanian population, despite a great resistance to the acceptance of Orthodox religion. The later Ottoman occupation spanning about five centuries resulted in conversion to Islam as the dominant religion. Because of this complex history, today the territory surrounding Prizren is occupied by diverse ethnic groups (Albanians, Serbs, Turks, Bosniaks, Gorani and Romani) and diverse religions (Muslim (Albanians, Turks, Bosniaks and Gorani), Catholic (Albanians) and Orthodox (Serbians)). The intercultural mixing of various communities in the same area has resulted in a dynamic form of TEK, with the impact of one traditional culture on another illustrated in both the uses and names of useful plants found in the local flora.

Before World War II, healthcare in this region was almost entirely based on traditional medicine, and these traditions continued after the war as well. Healthcare was commonly attended to within the family, and all physical and mental illnesses were treated with traditional medicines and rituals. These folk-medical traditions continue even now, especially in the more mountainous and isolated areas. Local people have withstood the extreme conditions of this region for centuries – including very harsh winters. Until very recent decades, limitations in infrastructure and communication forced local residents to be self-sufficient in the provision of their food and healthcare. As a result, their primary pharmacopoeia consisted of local medicinal plants.

Today, the residents southwest Kosovo are ethnic Albanians (who speak Gheg varieties of the Albanian language), Serbians (Serbian language), Turks (Turkish language), Bosniaks (Bosnian language), Gorani (Slavic language, Gora dialect or "Našinski" which is similar to Bosnian language) and Roma (Romani language). Regarding the population census conducted in 2011, there were 177,781 inhabitants in the Prizren municipality (145,718 Albanians, 237 Serbians, 9,091 Turks, 16,896 Bosniaks, 2,899 Roma, 1,350 Ashkali, 168 Egyptians, 655 Gorani and 386 others) and 33,997 in the Dragash municipality (20,287 Albanians, 7 Serbians, 202 Turks, 4,100 Bosniaks, 3 Roma, 4 Ashkali, 3 Egyptians, 8,957 Gorani, and 283 others) [33]. Population numbers and the ethnic structure of these municipalities have fluctuated over time due to the natural growth and the migration of the population. Most recently, local populations have been negatively affected by migration due to displacement and the harsh economic conditions caused by the last Kosovo War (1998–1999). The most common directions of migrations in Kosovo are from rural areas to urban areas and migration abroad. Migration patterns contribute to the rapid decline of traditional knowledge of plant species used as medicine, food and handicrafts; it has also contributed to a decline the vertical transmission of oral traditional knowledge from one generation to another. Small-scale farming and pastoral activities still represent the main economic income sources for the families in the study area. This is supplemented by remittances sent by relatives living in Germany or Switzerland, where the migrations of SW Kosovo were historically directed.

### The field study

**Figure 2:**
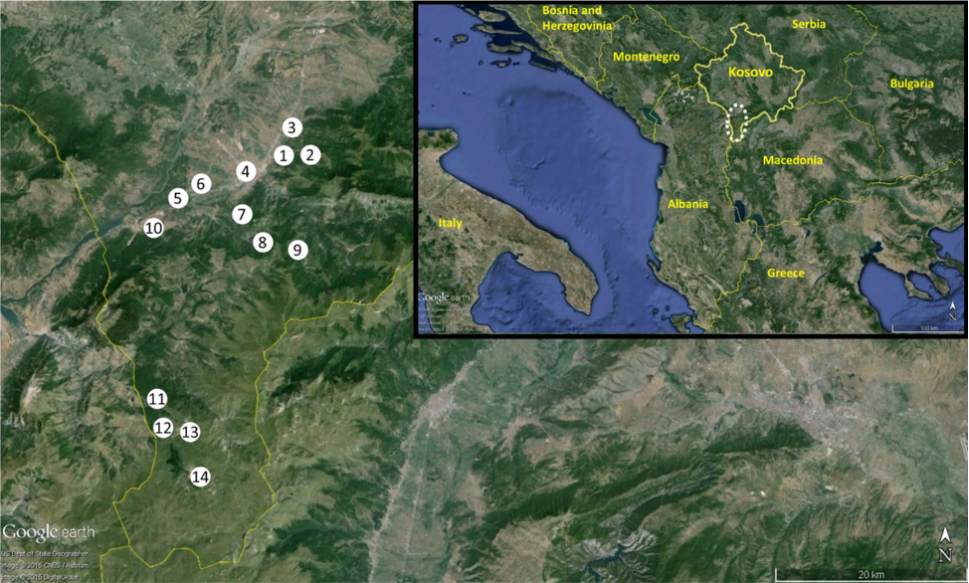
Map of study area and location of communities included in the study. Communities included in the study are indicated by number: 1) Gërnçar (710 m.a.s.l.), 2)Skorobisht (990 m), 3) Lubizhdë e Prizrenit (495 m), 4) Prizren (421 m), 5) Vlashnje (337 m), 6) Grazhdanik (385 m), 7) Leskovecë (830 m), 8) Lez (117 m), 9) Struzhë (1169 m), 10) Zhur (461 m), 11) Glloboçicë (1270 m), 12) Krushevë (1164 m), 13) Zlipotok (1395 m) and 14) Restelicë (1470 m).

Ethnobotanical field research was conducted in 14 villages belonging to the municipalities of Prizren (10 communities) and Dragash (4), located in Sharr Mountains, which are situated in the southern part of Kosovo (Figure 2). Field studies were conducted over a series of trips in 2012 and 2014. Snowball sampling methods were used to recruit informants and we particularly focused on local people who regularly use plants for medicinal purposes. Prior informed consent was obtained prior to conducting interviews and all researchers adhered to the ethical guidelines of the International Society of Ethnobiology [34].

TEK was recorded using semi-structured interviews. In particular, informal conversations were conducted around the issue of local plants traditionally used for food (wild food plants), medicine, and handicrafts. We sought in particular the following information: respondent name, age, gender, and community of residence; local botanical names of useful plants; plant part(s) used; preparation/administration details; local folk uses of plants. In total, data were collected from 139 informants: 61 Albanians (43 male, 18 female), 32 Bosniaks/Gorani (Bosniaks: 11 male, 7 female; Gorani: 10 male, 4 female) and 46 Turks (28 male, 18 female). With regards to the data analysis, data collected from the Bosniak and Gorani informants were merged as both are culturally similar and share the same religion and language. Gorani communities have been claimed by Bosniaks, Serbs, and Bulgarians and recently by Macedonians, but in Kosovo they are recognized as a distinct minority group.

The respondents were older than 50 years (with a few exceptions), mainly engaged in agricultural activities and typically inherited their ethnobotanical knowledge from their direct ancestors (parents, grandparents) via oral traditions. During the interviews, fresh plants were collected to create voucher specimens for the herbarium and whenever possible, informants were followed into the field to show us the quoted species. Most plant species were collected while flowering. Taxonomic identification was undertaken using relevant standard botanical literature of the area [35-38]. Plant nomenclature largely follows the *Flora Europaea* [39], while plant family assignments follow the current Angiosperm Phylogeny Group III guidelines [40]. Voucher specimens of the wild taxa were deposited at the Department of Biology (Herbarium code Pz/2013), University of Prishtina.

### Data analysis

#### Overlap analysis for cited taxa

**Figure 3:**
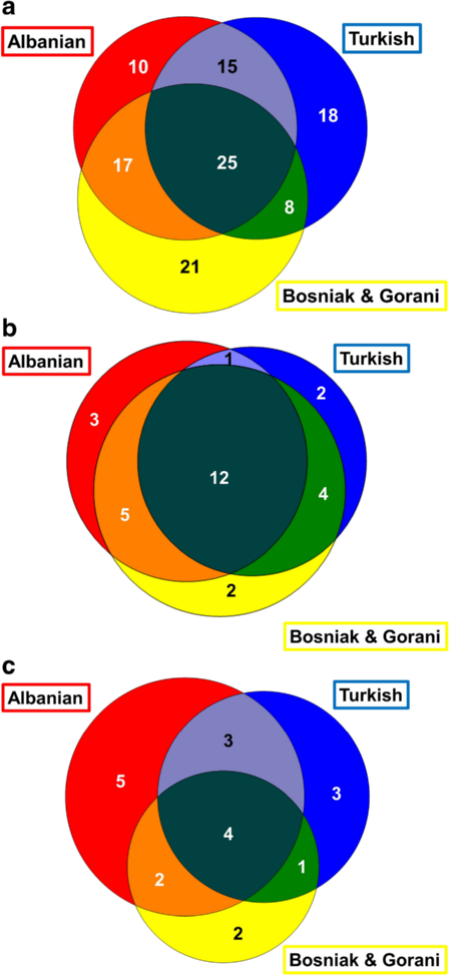
Venn diagram representing the overlap of taxa cited by Albanians, Bosniaks/Gorani, and Turks in the study area for: **a)** medicinal use, **b)** food use and **c)** handicraft use.

Taxa with use-citations based on general category of use (medicinal, food or handicraft) were compared across three groups (Albanian, Turks and Bosniaks/Gorani). Data are represented in the form of a Venn Diagram (Figure 3) to illustrate overlaps in use of taxa.

### Use-value for individual species

The use-value citation (*UV*_*c*_) index was calculated for each species for each ethnic group [41]. Here, we modified this method to calculate UV values in three different categories of use: medicinal, food, and handicraft. This index is useful for examination of relative importance of each species for a general category of use based on the number of use-citations. Briefly, it was calculated as follows:

$$ U{V}_c=\frac{{\displaystyle \sum }{N}_{uc}}{N} $$

Where N_uc_ is the number of use citation reports concerning a given species in a use category (e.g. medicinal, food, handicraft), divided by the total number of informants (N) in a specific group (e.g. Albanian, Turkish, or Bosniak & Gorani). In a recent paper by Quave and Pieroni [42], UV values were plotted on a two-dimensional matrix framework to assess relative values for individual species between two ethnic groups. Here, we expand upon this concept and apply it to a three-dimensional matrix for comparison of plant use-values for individual species between three ethnic groups that share access to the same environmental and botanical resources.

#### Three-dimensional (3-D) use-value matrix design and analysis

**Figure 4:**
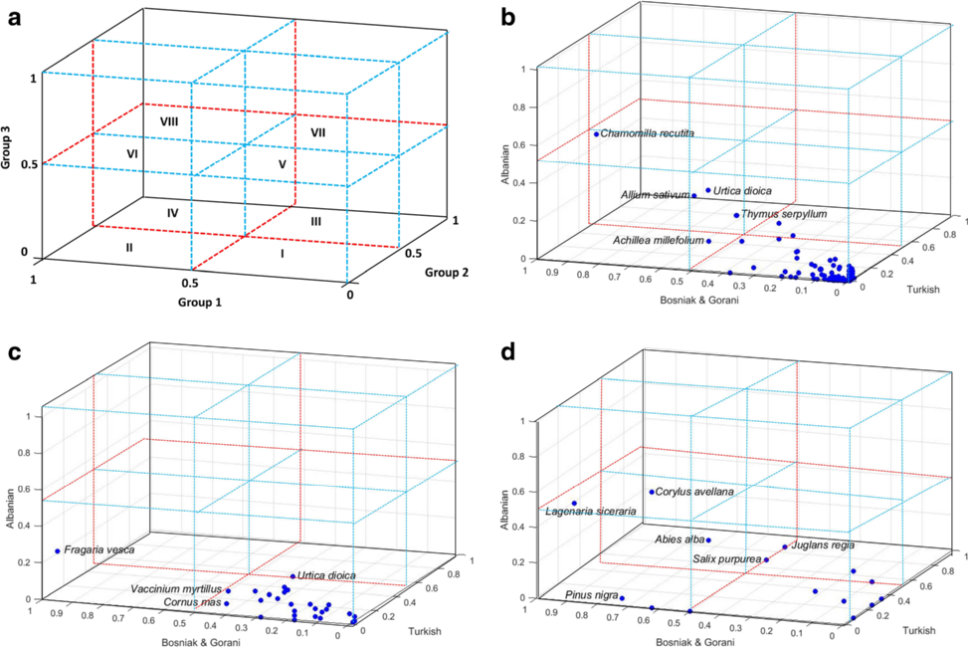
Use-value matrix comparison of three groups with access to the same flora and ecological resources. **a)** Matrix design. Each quadrant corresponds to a specific relationship concerning the adjusted plant use-value (details provided in the methods). Adjusted use-values (UV_adj_) are represented for cited taxa by ethnic group and general category **b)** medicinal use, **c)** food use and **d)** handicraft use.

We propose a new approach for the comparative analysis of how use-values differ in three ethnic groups, and across different general categories of use. The UV_c_ data for each category of use (medicinal, food, handicraft) were normalized to allow for comparison on a scale of 0–1. This was achieved by identifying the maximum UV_c_ value for each category of use (UV_max_). The UV_c_ for each species (and ethnic group) was then divided by the UV_max_ to create the adjusted UV value (UV_adj_) and plotted onto a 3-D scatterplot using MATLAB® software. Eight 3-D overlay quadrants were created to assist in classifying the UV_adj_ clusters (Figure 4A). They were defined as follows in relationship to the three ethnic groups being compared (Group 1: Bosniak/Gorani; Group 2: Turkish; Group 3: Albanian):

- Quadrant I: Taxa with UV_adj_ ≤0.05 for all three groups, indicating consensus in low use-value across groups.
- Quadrant II: Group 1 UV_adj_ > 0.05; Group 2 UV_adj_ ≤0.05; Group 3 UV_adj_ ≤0.05, indicating consensus on lower use-value among Group 2 and 3, but higher use-value for Group 1.
- Quadrant III: Group 1 UV_adj_ ≤0.05; Group 2 UV_adj_ >0.05; Group 3 UV_adj_ ≤0.05, indicating consensus on lower use-value among Group 1 and 3, but higher use-value for Group 2.
- Quadrant IV: Group 1 UV_adj_ >0.05; Group 2 UV_adj_ >0.05; Group 3 UV_adj_ ≤0.05, indicating consensus on higher use-value among Group 1 and 2, but lower use-value for Group 3.
- Quadrant V: Group 1 UV_adj_ ≤0.05; Group 2 UV_adj_ ≤0.05; Group 3 UV_adj_ >0.05, indicating consensus on lower use-value among Group 1 and 2, but higher use-value for Group 3.
- Quadrant VI: Group 1 UV_adj_ >0.05; Group 2 UV_adj_ ≤0.05; Group 3 UV_adj_ >0.05, indicating consensus on higher use-value among Group 1 and 3, but lower use-value for Group 2.
- Quadrant VII: Group 1 UV_adj_ ≤0.05; Group 2 UV_adj_ >0.05; Group 3 UV_adj_ >0.05, indicating consensus on higher use-value among Group 2 and 3, but lower use-value for Group 1.
- Quadrant VIII: Taxa with UV_adj_ >0.05 for all three groups, indicating consensus in high use-value across groups.

Quadrant assignments are also reported in Tables 1, 2 and 3.

**Table 1:** **Medicinal plant used in the study area**

**Botanical taxon, family and voucher specimen code**	**Status**	**Folk name(s)** ^**a**^	**Part(s) used**	**Administration**	**Treated disease(s) or folk medical uses(s)**	**Alb N**_**uc**_^**b**^	**Bo/Go N**_**uc**_^**b**^	**Tur N**_**uc**_^**b**^	**UV**_**Alb**_^**c**^	**UV**_**Bo/Go**_^**c**^	**UV**_**Tur**_^**c**^	**Q** ^**d**^
*Abies alba* Mill. (Pinaceae) 14/Pz/2013	W	Bredhi^ALB^	Wood	Resin, mixed with fat	Anti-fungal	2	0	0	0.033	0	0	I
*Agrimonia eupatoria* L*.* (Rosaceae) 08/Pz/2013	W	Petrovac^BOG^	Aerial parts	Infusion	Anti-allergic,	0	3	1	0	0.219	0.065	I
		Kezell japrak^TUR^			Earache,	0	1	0				
					Anti-inflammatory,	0	2	2				
					Anti-diarrheal	0	1	0				
*Agropyron repens* (L.) Beauv. (Poaceae) 07/Pz/2013	W	Bari i magarit^ALB^	Aerial parts	Infusion	Anti-hemorrhoidal,	2	0	0	0.049	0.125	0.065	I
		Priovina^ALB^			Respiratory system disorders,	0	3	0				
		Priovina^BOG^										
					Urinary tract disorders	1	1	0				
*Alchemilla vulgaris* L. (Rosaceae) 05/Pz/2013	E	Alhemıla^ALB^	Aerial parts	Infusion	Improve fertility in women	2	0	0	0.033	0	0	I
*Achillea millefolium* L*.* (Asteraceae) 03/Pz/2013	W	Barëpezmatimi^ALB^	Aerial parts	Infusion	Anti-cholesterolemic,	0	4	0	0.557	2.656	0.652	I
		Hajdutska trava^BOG^			Anti-coagulant,	3	6	0				
		Hajdut oti^TUR^			Appetizing,	5	3	6				
					Anti-microbial,	12	28	8				
					Antiemetic,	2	0	0				
					Carminative and spasmolytic,	7	31	11				
					Anti-diabetic,	0	4	1				
					Antacid,	0	1	0				
					Menstrual pains,	0	1	0				
					Influenza,	2	5	3				
					Stomachache	3	2	1				
*Allium cepa* L*.* (Amaryllidaceae) 11/Pz/2013	C	Kepa^ALB^	Bulbs	Eaten raw,	Anti-cholesterolemic	3	7	1	0.656	1.125	0.5	I
		Cerveni luk^BOG^		Topically in wound	Anti-bacterial	37	29	22				
		Kepi^TUR^										
*Allium porrum* L*.* (Amaryllidaceae) 09/Pz/2013	C	Purri^ALB^	Leaves	Eaten	Thyroid disorders	2	4	0	0.033	0.125	0	I
		Prazılluk^BOG^										
*Allium sativum* L*.* (Amaryllidaceae) 10/Pz/2013	C	Hudra^ALB^	Bulbs	Eaten	Anti-hypertensive,	16	24	11	1.361	3.188	1.63	II
		Beli lluk^BOG^ Sarimsak^TUR^			Anti-fungal,	11	8	3				
					Anti-ageing,	0	3	5				
					Urinary tract infections,	2	5	3				
					Anti-hypertensive,	18	14	12				
					Bronchitis							
						12	6	8				
				Mixed with honey	Bronchitis,	14	7	7				
					Anti-tussive,	2	8	3				
					Skeletal system enhancement,	0	2	2				
					Immunostimulant,	0	5	3				
					Anti-anemic,	0	2	1				
					Respiratory system disorders,	8	17	13				
					Skin regeneration	0	1	4				
*Althaea officinalis* L. (Malvaceae)	W	Mullaga^ALB^	Flowers	Infusion	Anti-tussive/expectorant	12	18	9	0.197	0.563	0.196	I
		Beli slez^BOG^										
		Gul hatem^TUR^										
*Aloe vera* (L.) Burm.f. (Xanthorrhoeaceae) 06/Pz/2013	C	Aloa^ALB^	Leaves	Eaten fresh with honey	Anti-tumor	3	0	0	0.049	0	0	I
*Amanita caesarea* (Scop.) Pers. (Amanitaceae)	W	Kërpurdha^ALB^	Fruiting body	Topically applied	Skin infections	2	0	0	0.033	0	0	I
*Apium graveolens* L*.* (Apiaceae) 12/Pz/2013	C	Kereviz^TUR^	Aerial parts	Infusion	To treat sterility	0	0	2	0	0	0.109	I
			Roots	Infusion	Diuretic, appetizing	0	0	3				
*Arctostaphylos uva-ursi* (L.) Spreng. (Ericaceae) 02/Pz/2013	W	Çaj uvin^ALB^	Leaves	Infusion	Urinary tract infections	12	16	0	0.344	1	0	I
		Rrush arushe^ALB^	Aerial parts	Infusion	Urinary tract infections and pains	9	16	0				
		Medvegje ushi^BOG^										
*Artemisia absinthium* L*.* (Asteraceae) 01/Pz/2013	W	Fshisa^ALB^	Leaves	Infusion	Anti-anemic, Anti-malarial	2	3	0	0.377	1.25	0	I
		Pelini^ALB^				0	2	0				
		Divli pelin^BOG^	Aerial parts	Infusion	Anti-diabetic,	0	2	0				
					Appetizing,	4	5	0				
					Improve hormonal balance in women,	0	2	0				
					Anti-parasitic,	1	1	0				
					Relaxant, stomachache	12	16	0				
			Fruits	Infusion	Lithontriptic,	0	1	0				
					Anti-asthmatic,	2	3	0				
					Anti-diabetic							
						2	5	0				
*Avena sativa* L. (Poaceae) 15/Pz/2013	C	Thekna^ALB^	Aerial parts	Infusion	Skeletal system enhancement	2	0	4	0.033	0	0.087	I
		Jullaf ^TUR^										
*Betula alba* L. (Betulaceae) 16/Pz/2013	W	Mështekna^ALB^		Infusion	Diuretic,	0	1	0	0.164	0.563	0.217	I
		Plep i bardhë^ALB^			Edema,	0	2	2				
		Breza^BOG^			Urinary disorders	2	4	2				
		Hush agagji^TUR^		Infusion used for hair wash	Alopecia	8	11	6				
*Brassica rapa* L. (Brassicaceae)	C	Rrepa^ALB^ Shalgam^TUR^	Taproot	Eaten	Eye disorders, Immunostimulant	4	0	3	0.066	0	0.065	I
*Calendula officinalis* L*.* (Asteraceae) 27/Pz/2013	C	Lule dukati^ALB^ Neven^BOG^	Aerial parts	Extracted with different oils	Anti-bacterial, anti-fungal,	0	14	0	0	0.938	0	I
					Vulnerary for burn wounds and sunburns	0	16	0				
*Capsella bursa-pastoris* (L.) Medik. (Brassicaceae) 28/Pz/2013	C	Më do s’më don^ALB^ Tarqushak^BOG^	Aerial parts	Infusion	Anticoagulant	2	5	0	0.033	0.156	0	I
		Hoqunequ^BOG^										
*Castanea sativa* Mill*.* (Fagaceae) 19/Pz/2013	W/C	Gështaja^ALB^	Flowers	Infusion	Anti-anemic,	3	0	0	0.131	0	0	I
					Bronchitis,	2	0	0				
					Anti-tussive	2	0	0				
			Cortex	Infusion	Anti-tussive	1	0	0				
*Centaurium erythraea* Rafn (Gentianaceae) 29/Pz/2013	W	Kantarioni i kuq^ALB^	Aerial parts	Infusion	Anticoagulant,	0	2	0	0.426	1.063	0.391	I
		Bari i etheve^ALB^			Anti-pyretic,	13	18	9				
		Kicica^BOG^			Anti-malarial,	9	12	6				
					Appetizing,	2	1	0				
					Anti-anemic,	0	1	0				
					Antacid,	0	0	1				
					Immunostimulant	2	0	2				
*Centaurea cyanus* L. (Asteraceae) 20/Pz/2013	W	Kokoçeli^ALB^	Flower	Infusion	Respiratory disorders	3	1	0	0.049	0.031	0	I
		Kicica^BOG^										
*Chamomilla recutita* (L.) Rauschert (Asteraceae) 43/Pz/2013	W	Lule qeni^ALB^	Aerial parts	Infusion	Antimicrobial,	31	37	25	3.115	5.5	2.543	VI
		Kamomil^ALB^			Infections of the digestive tract,	12	10	0				
		Kamilica^BOG^			Urinary tract infections,	20	6	13				
					Eye infections	11	8	2				
		Babunec^BOG^				25	15	17				
		Papatja^TUR^										
		Sari çiçek^TUR^										
			Flowers	Infusion	Anti-tussive,	14	26	22				
					Anti-bacterial,	31	35	12				
					Influenza,	11	9	9				
					Oral cavity infections,	9	3	4				
					Anti-hemorrhoidal,	1	0	7				
					Alopecia,	2	0	0				
					Wound healing,	20	23	6				
					Relaxant	3	4	0				
*Chelidonium majus* L. (Papaveraceae) 30/Pz/2013	W	Tamelqak^ALB^	Latex	Topically used	Skin infections, warts	11	0	7	0.18	0	0.152	I
		Kena qıqegı^TUR^										
*Cichorium intybus* L. (Asteraceae) 21/Pz/2013	W	Cikorja^SHQ^	Aerial parts	Infusion	Hepatic disorders	2	0	4	0.033	0	0.087	I
		Mavi çiçek^TUR^										
		Satali bitki ^TUR^										
*Citrus limon* (L.) Osbeck (Rutaceae) 31/Pz/2013	*C*	Limun^BOG^	Fruits	Lemon juice mixed with sugar	Anti-tussive, bronchitis	0	6	3	0	0.188	0.065	I
		Limon^TUR^										
*Cornus mas* L. (Cornaceae) 23/Pz/2013	W	Thana^ALB^	Fruits	Infusion	Anti-anemic,	0	5	1	0.148	0.656	0.304	I
		Drenilje^BOG^			Anti-hypertensive,	5	3	6				
		Dirnina^TUR^			Anti-diarrheal,	0	4	2				
					Anti-malarial,		1	1				
					Anti-emetic in early stage of	2						
					pregnancy (for morning sickness),		5	2				
					Improve immunity,	2						
					Anti-hemorrhoidal	0	2	1				
							1	1				
*Corylus avellana* L. (Betulaceae) 24/Pz/2013	W	Lejthı^ALB^	Leaves	Infusion	Anti-tussive,	0	2	1	0	0.18	0.022	I
		Adi findik^TUR^			Antacid,	0	3	0				
					Hepatic disorders	0	1	0				
*Cotinus coggygria* Scop. (Anacardiaceae) 64/Pz/2013	W	Ruj^TUR^	Leaves	Infusion	Stomach disorders,	0	0	2	0	0	0.174	I
		Boyaci sumak^TUR^			Kidney disorders,	0	0	1				
					Anti-diarrheal	0	0	4				
			Fruits	Infusion	Anti-diarrheal	0	0	1				
*Crataegus monogyna* Jacq. (Rosaceae) 18/Pz/2013 05/Pz/11	W	Murrizi^ALB^	Fruits	Infusion	Improve blood circulation,	27	21	18	1.279	2.406	1.174	I
		Gllog^BOG^			Anti-hypertensive,	22	20	12				
		Adi aliç^TUR^			Neurorelaxant	2	6	1				
			Leaves and flowers	Infusion	Anti-hypertensive,	12	20	12				
					Anti-diabetic,	2	6	2				
					Anti-cholesterolemic	13	4	9				
*Cucumis sativus* L. (Cucurbitaceae) 26/Pz/2013	C	Kastravec^ALB^	Fruits and seeds	Eaten fresh	Kidney disorders,	4	3	0	0.131	0.375	0	I
		Kastravac^BOG^			Improve blood circulation,	3	7	0				
					Improve skin vitality,	1	1	0				
					Eye disorders	0	1	0				
*Cucumis melo* L*.* (Cucurbitaceae) 25/Pz/2013	C	Pjepni^ALB^	Fruits	Eaten fresh	Infection of digestive system	4	0	0	0.066	0	0	I
*Cydonia oblonga* Mill. (Rosaceae)	C	Ftua^ALB^	Leaves	Infusion	Anti-diarrheal	7	4	0	0.115	0.125	0	I
		Dunja^BOG^										
*Dryopteris filix*-*mas* (L.) Schott (Dryopteridaceae)	W	Paprat^BOG^	Leaves	Infusion	Anti-parasitic	0	2	0	0	0.063	0	I
*Equisetum arvense* L. (Equisetaceae) 07/Pz/11	W	Konksi rep^BOG^	Aerial parts	Infusion	Hepatic disorders,	0	2	0	0	0.281	0	I
					Kidney infections and pain	0	7	0				
*Euphorbia amygdaloides* L. (Euphorbiaceae)	W	Mali mleq^BOG^	Latex	Topically used	Warts	0	3	0	0	0.094	0	I
*Foeniculum vulgare* Mill*.* (Apiaceae) 32/Pz/2013	W	Kopër^ALB^ Rezene^BOG^	Fruits	Infusion	Eye disorders,	0	2	0	0.197	0.375	0.130	I
		Anason^TUR^			Galactogogue,	0	1	1				
					Spasmolytic	12	9	5				
*Fragaria vesca* L. (Rosaceae)	W	Dredhza^ALB^	Fruits	Infusion	Digestive, Spasmolytic	2	0	3	0.082	0	0.174	I
		Divla jagoda^BOG^				3	0	5				
*Fumaria officinalis* L. (Papaveraceae) 33/Pz/2013	W	Shatere^TUR^	Aerial parts	Infusion	Diuretic,	0	2	3	0	0.188	0.087	I
					Relaxant,	0	1	1				
					Anti-hypertensive	0	3	0				
*Galium verum* L. (Rubiaceae) 35/Pz/2013	W	/	Aerial parts	Infusion	Kidney disorders,	0	0	2	0	0	0.130	I
					Skin regeneration	0	0	4				
*Gentiana lutea* L*.* (Gentianaceae) 34/Pz/2013	W	Geciana^ALB^	Roots	Infusion	Digestive disorders,	5	12	0	0.180	0.375	0	I
		Lincura^BOG^			Flavor additive for alcoholic beverage	6	0	0				
*Geranium sanguineum* L. (Geraniaceae)	W	Zdrvac^BOG^	Aerial parts	Infusion	Respiratory disorders, laryngitis	0	3	0	0	0.094	0	I
*Helianthus annuus* L*.* (Asteraceae)	C	Lule djellı^ALB^	Seeds	Extracted with animal fat	Skin infections	6	5	0	0.098	0.156	0	I
		Suncokret^BOG^										
*Hordeum vulgare* Jess. (Poaceae)	C	Elbi^ALB^	Seeds	Flour, mixed with oil	Wound healing	5	0	2	0.082	0	0.043	I
		Jeçmenik^TUR^										
		Arpa elbi^TUR^										
*Humulus lupulus* L. (Cannabaceae) 37/Pz/2013	W	Bari sherbetit^ALB^	Aerial parts	Infusion	Insomnia,	3	3	1	0.180	0.563	0.217	I
		Amel brumit^TUR^			Appetizing,	2	5	2				
		Amel^TUR^			Neurorelaxant	0	2	2				
			Fruits	Infusion	Insomnia,	6	5	3				
					Diuretic,	0	3	1				
					Prostate disorders	0	0	1				
*Hypericum perforatum* L. (Hypericaceae) 36/Pz/2013 08/Pz/11	W	Kantarioni^ALB^	Aerial parts	Infusion	Anti-anemic, Wound healing, Anticoagulant, Neurorelaxant, Antacid	0	4	0	0.475	1.844	0	I
		Kantarion^BOG^				12	32	0				
						0	1	0				
						16	21	0				
						1	1	0				
*Inula* sp. (Asteraceae)	W	Omani^TUR^	Roots	Infusion	Anti-tussive, Bile simulation, Diuretic	0	0	2	0	0	0.130	I
		Safra oti^TUR^				0	0	3				
						0	0	1				
*Juglans regia* L. (Juglandaceae) 40/Pz/2013	C	Arra^ALB^	Fruits	Eaten	Anti-parasitic, Thyroid disorders	3	0	0	0.115	0	0	I
						4	0	0				
*Juncus effusus* L. (Juncaceae)	W	Xuklla^TUR^	Aerial parts	Infusion	Urinary tract disorders	0	0	3	0	0	0.065	I
*Juniperus communis* L. (Cupressaceae) 39/Pz/2013	W	Gëllija^ALB^	Wood	Extracted with oil, topically used in skin	Anti-fungal, Skin depigmentation	6	17	5	0.623	2.094	0.696	I
		Kleka^BOG^				2	11	2				
		Ardeq^TUR^										
			Fruits	Extracted with alcohols	Anti-rheumatic	5	3	0				
				Infusion	Tuberculosis,	6	2	9				
					Anti-rheumatic,	3	11	5				
					Lithontriptic,	9	21	11				
					Anti-asthmatic,	4	1	0				
					Anti-diabetic	3	1	0				
*Lactuca sativa* L. (Asteraceae) 41/Pz/2013	C	Sallata^ALB^	Aerial parts	Eaten fresh	Headache,	0	0	2	0	0	0.043	I
		Marrolli^TUR^			Galactogogue	0	0	4				
*Leonurus cardiaca* L. (Lamiaceae)	W	Ayslan kuyrgu^TUR^	Aerial parts	Infusion	Cardiotonic,	0	0	3	0	0	0.065	I
					Improve blood circulation,	0	0	2				
					Memory enhancement	0	0	4				
*Lycoperdon* sp. (Agaricaceae) 60/Pz/2013	W	Pufka^ALB^	Powder	Topically applied	Wound healing, Hemostatic	4	1	0	0.066	0.031	0	I
		Mantari^BOG^				8	4	0				
*Lycopodium clavatum* L*.* (Lycopodiaceae)	W	Bari qibritit^ALB^	Aerial parts	Topically applied to skin	Anti-microbial	2	0	0	0.033	0	0	I
				Infusion	Hepatitis	1	0	0				
*Malva sylvestris* L. (Malvaceae) 44/Pz/2013	W	Mullaga^ALB^	Aerial parts	Extracted with fat (*melhem*)	Wound healing,	3	5	2	0.262	0.250	0.283	I
		Mali slez ^BOG^			Anti-acne	5	2	0				
		Ebe gumeci^TUR^										
			Flowers	Infusion	Anti-tussive,	2	1	3				
					Bronchitis,	2	0	4				
					Antimicrobial	4	0	4				
*Mespilus germanica* L. (Rosaceae) 47/Pz/2013	C	Mushmolla^ALB^	Aerial parts	Infusion	Anti-diarrheal,	4	0	0	0.115	0	0	I
					Anti-diabetic,	2	0	0				
					Ear disorders	1	0	0				
*Melissa officinalis* L*.* (Lamiaceae) 42/Pz/2013	W	Bari i bletës^ALB^	Aerial parts	Infusion	Neurorelaxant,	3	8	5	0.475	0.406	0.413	I
		Matoqına^BOG^			Headache,	3	1	4				
		Molshvatrava^BOG^			Anti-hypertensive,	2	0	1				
					Appetizing,	6	0	1				
					Improve blood circulation,							
					Bronchitis,	3	0	1				
					Anti-anemic,	7	2	3				
					Anti-hallucinogenic,	0	1	3				
					Respiratory disorders	5	1	1				
*Mentha longifolia* (L.) Huds. (Lamiaceae) 45/Pz/2013	C	Çaj nana^ALB^ Nana^BOG^	Aerial parts	Infusion	Stomach disorders,	0	3	0	0.311	0.75	0	I
					Carminative,	3	4	0				
					Influenza,	2	6	0				
					Respiratory system infections,	8	9	0				
					Anti-tussive,	4	1	0				
					Expectorant	2	1	0				
*Mentha pulegium* L. (Lamiaceae) 46/Pz/2013	W	Divla menta^BOG^	Aerial parts	Infusion	Neurorelaxant,	0	3	0	0	0.625	0	I
					Improve blood circulation,	0	7	0				
					Respiratory system infections,	0	9	0				
					Antitussive	0	1	0				
*Momordica charantia* L. (Cucurbitaceae) 50/Pz/2013	C	Kudret nare^TUR^	Fruits	Mixed with oil –internal use	Wound healing,	0	0	7	0	0	0.435	I
		Sari kadak^TUR^			Anti-diabetic,	0	0	1				
					Anti-cancer	0	0	4				
				Mixed with oil - topically applied	Vulnerary for burn wounds	0	0	8				
*Morus alba* L. (Moraceae) 49/Pz/2013	C	Mani i bardhe^ALB^	Leaves	Infusion	Anti-diabetic	0	0	4	0	0	0.087	I
		Akdut^TUR^										
*Morus nigra* L*.* (Moraceae) 48/Pz/2013	C	Mani i zi^ALB^	Fruits	Eaten fresh	Infections of upper part of respiratory system	3	0	5	0.148	0	0.196	I
		Dut^TUR^										
		Karadut^TUR^										
			Leaves	Infusion	Anti-pyretic, Diuretic	6	0	4				
*Ocimum basilicum* L. (Lamiaceae) 51/Pz/2013	C	Bosiljak^BOG^	Aerial parts	Infusion	Carminative,	0	3	0	0	0.219	0	I
					Kidney infections,	0	1	0				
					Tuberculosis	0	3	0				
*Olea europaea* L. (Oleaceae) 15/Pz/11	C	Ullini^ALB^	Fruits	Eaten fresh	Tuberculosis, Spasmolytic	0	1	0	0.131	0.219	0.283	I
		Maslina^BOG^				6	5	2				
		Zejtın tanesi^TUR^										
			Leaves	Infusion	Improve blood circulation,	0	1	5				
					Anti-diabetic,	1	0	3				
					Anti-hypertensive	1	0	3				
*Orchis morio* L. (Orchidaceae) 53/Pz/2013	W	Salep^BOG^	Tubers	Infusion	Influenza,	0	2	1	0	0.219	0.043	I
		Sahlep^TUR^			Stomach disorders,	0	1	1				
					Wound healing	0	4	0				
*Origanum vulgare* L. (Lamiaceae) 52/Pz/2013	W	Çaj mali^ALB^	Aerial parts	Infusion	Anti-tussive,	6	4	2	0.279	0.750	0.304	I
		Origano^BOG^			Influenza,	2	3	1				
		Toqilla^TUR^			Respiratory system infections	9	17	11				
*Petroselinum crispum* (Mill.) Fuss (Apiaceae) 61/Pz/2013	C	Majdanoz^ALB^	Aerial parts	Infusion	Anti-cholesterolemic	2	0	4	0.148	0	0.304	I
		Magdenoz^TUR^			Anti-diabetic,	4	0	1				
					Galactogogue,	2	0	5				
					Anticoagulant	1	0	4				
*Phaseolus vulgaris* L. (Fabaceae) 62/Pz/2013	C	Pasul^ALB^	Aerial parts	Infusion	Anti-diabetic	2	0	3	0.033	0	0.065	I
		Jer pasul^TUR^										
*Pimpinella anisum* L. (Apiaceae)	C	Bati i gjinit^ALB^ Anason^TUR^	Aerial parts	Infusion	Spasmolytic,	0	0	5	0	0	0.283	I
					Carminative,	0	0	5				
					Anti-ageing,	0	0	2				
					Galactogogue	0	0	1				
*Pinus nigra* J.F. Arnold*.* (Pinaceae)	W/C	Pisha^ALB^	Resin	Extracted with oil	Skin infections	3	0	6	0.049	0	0.130	I
		Kara qam^TUR^										
*Plantago major* L*.* (Plantaginaceae) 54/Pz/2013	W	Dejzi^ALB^	Leaves	Infusion	Wound healing	0	6	0	0	0.313	0	I
		Bokvica^BOG^	Arial parts	Infusion	Skin infections	0	3	0				
		Zenska bokvica^BOG^	Flowers	Infusion	Digestive and urinary disorders	0	1	0				
*Polygonum aviculare* L. (Polygonaceae)	W	Barthek^ALB^	Aerial parts	Infusion	Urinary system disorders,	2	0	4	0.082	0	0.109	I
		Kusekmezi^TUR^			Anti-coagulant	3	0	1				
		Troket^TUR^										
*Populus alba* L*.* (Salicaceae)	W	Plepi^ALB^	Aerial parts	Topically uses	Wound healing	0	0	3	0	0	0.087	I
		Ak kavak^TUR^	Leaves	Infusion	Urinary tract disorders	0	0	1				
		Beyaz kavak^TUR^										
*Primula veris* L*.* (Primulaceae) 56/Pz/2013	W	Myzhdja e pranverës^ALB^	Flowers	Infusion	Headache,	0	2	1	0.508	1.094	0.543	I
		Jaglika^BOG^			Anti-tussive,	11	14	9				
		Zuti vet^BOG^			Respiratory system disorders,	14	7	9				
		Jagorcevina^BOG^			Improve blood circulation	3	1	1				
		Quha çicegi^TUR^										
			Aerial parts	Infusion	Anti-tussive,	2	4	0				
					Expectorant,	1	6	4				
					Bronchitis	0	1	1				
*Prunus avium* L. (Rosaceae)	C	Qershia^ALB^	Fruits	Decoction	Anti-hypertensive,	2	0	0	0.262	0	0	I
					Improve blood circulation,	5	0	0				
					Anti-bacterial,	3	0	0				
					Digestive tract disorders	1	0	0				
			Resin	Topically used	Scabies	5	0	0				
*Prunus domestica* L. (Rosaceae) 55/Pz/2013	C	Sljiva^BOG^	Fruits	Decoction	Hepatic disorders,	0	1	0	0	0.438	0	I
					Anti-hemorrhoidal,	0	5	0				
					Anti-parasitic,	0	2	0				
					Constipation	0	6	0				
*Prunus spinosa* L. (Rosaceae)	W	Kulumrija^ALB^	Flowers	Infusion	Constipation	0	3	1	0.131	0	0.065	I
		Ternina^BOG^	Fruits	Infusion	Anti-diabetic,	7	2	4				
		Kurumlia^TUR^			Hepatic disorders	0	1	1				
			Leaves	Infusion	Improve digestion,	1	1	2				
					Appetizing	0	2	1				
*Pteridium aquilinum* (L.) Kuhn. (Dennstaedtiaceae)	W	Firi^ALB^	Leaves	Extracted with oil	Wound healing	0	0	3	0	0	0.065	I
		Qiban otu^TUR^										
*Pulmonaria officinalis* L*.* (Boraginaceae)	W	Bar ı mushkerıve^ALB^ Pluqnjak^BOG^	Aerial parts	Infusion	Anti-tussive,	1	3	0	0.016	0.125	0	I
					Bronchitis	0	1	0				
*Pyrus communis* L. (Rosaceae) 58/Pz/2013	W	Dardha^ALB^	Fruits	Infusion	Cardiotonic,	0	3	0	0	0.125	0.043	I
		Dardha eger^ALB^			Hepatic disorders	0	1	2				
		Armut^TUR^										
*Raphanus sativus* L. (Brassicaceae) 63/Pz/2013	C	Rotkva^BOG^	Taproot	Infusion	Digestive system infections,	0	2	0	0	0.375	0	I
		Cvekla^BOG^			Bronchitis,	0	4	0				
					Anti-anemic,	0	1	0				
					Anti-rheumatic	0	5	0				
*Ribes rubrum* L. (Grossulariaceae)	C	Ribizla^BOG^	Fruits	Infusion	Anti-rheumatic,	0	3	0	0	0.344	0	I
					Anti-malarial,	0	1	0				
					Anti-allergic,	0	2	0				
					Heart disorders	0	5	0				
*Robinia pseudoacacia* L*.* (Fabaceae) 68/Pz/2013	W	Bagremi^ALB^	Flowers	Infusion	Skin infections	3	0	2	0.049	0	0.043	I
		Akasya^TUR^										
*Rosa canina* L*.* (Rosaceae) 67/Pz/2013	W	Kaça^ALB^	Fruits	Infusion	Improve immunity,	3	1	0	0.328	0.594	0	I
		Shipak^BOG^			Hepatic disorders,	1	2	0				
		Sipurak^BOG^			Anti-anaemic,	1	5	0				
					Influenza,	6	3	0				
					Digestive tract disorders.	9	8	0				
*Rubia tinctorum* L. (Rubiaceae)	W	Crvenka^BOG^	Aerial parts	Infusion	Kidney disorders,	0	3	0	0	0.281	0	I
					Skeletal disorders, tuberculosis	0	1	0				
					“*Saraxha*” (cutaneous tuberculosis)	0	5	0				
*Rubus fruticosus* L. (Rosaceae) 65/Pz/2013	W	Mana^ALB^	Aerial parts	Infusion	Anti-anemic,	0	3	0	0	1.031	0	I
		Kupina^BOG^			Improve blood circulation,	0	1	0				
					Anti-hypertensive,	0	4	0				
					Wound healing	0	3	0				
					Anti-diabetic,	0	4	0				
					Antimycotic	0	1	0				
			Fruits	Infusion	Anti-anemic,	0	4	0				
					Anti- diarrheal,	0	1	0				
					Kidney infections,	0	2	0				
					Oral cavity infections,	0	5	0				
					Hypertensive,	0	2	0				
					Anti-parasitic,	0	1	0				
					Anti-tussive	0	2	0				
*Rubus idaeus* L. (Rosaceae) 66/Pz/2013	W	Mjedra^ALB^	Leaves	Infusion	Improve blood circulation,	0	5	0	0	1.188	0	I
		Malina^BOG^			Anti-hypertensive,	0	5	0				
					Anti-diarrheal,	0	3	0				
					Anti-tussive,	0	2	0				
					Anti-pyretic,	0	1	0				
					Oral cavity infections	0	5	0				
			Roots	Infusion	Anti-hypertensive,	0	3	0				
					Wound healing	0	4	0				
			Fruits	Infusion	Dysentery,	0	1	0				
					Tonsillitis,	0	3	0				
					Digestive disorders	0	2	0				
			Flowers	Extracted with olive oil	To treated skin wounds caused by insects and snakes	0	4	0				
*Salix alba* L. (Salicaceae) 70/Pz/2013	W	Vrba^BOG^	Leaves	Infusion	Hepatic disorders	0	3	0	0	0.313	0	I
			Cortex	Infusion	Antipyretic,	0	5	0				
					Analgesic	0	2	0				
*Salvia officinalis* L*.* (Lamiaceae)	C	Zalfija^BOG^	Aerial parts	Infusion, then added honey	Tonsillitis and other infection of respiratory system,	0	7	0	0	0.344	0	I
					Anti-diabetic	0	2	0				
					Antiperspirant	0	2	0				
*Sambucus nigra* L*.* (Adoxaceae) 69/Pz/2013	W	Shtogu^ALB^	Flowers	Infusion	Bronchitis,	14	7	15	0.787	1.250	0.891	I
		Zova^BOG^			Anti-tussive,	8	5	3				
		Bos zova^BOG^			Expectorant, Antiperspirant,	3	7	8				
		Murver^TUR^			Anti-halitosis,	2	1	4				
		Forboz^TUR^			Influenza,	0	1	4				
					Anti-asthmatic,	6	8	2				
					Stomach disorders,	9	4	1				
					Urinary tract disorders	4	1	1				
						1	1	1				
				Extracted with fish oil	Anti-anemic, Improve immunity	0	3	1				
				Extracted with oil – topically used	Vulnerary for burns, skin infections	1	2	1				
*Satureja montana* L. (Lamiaceae) 19/Pz/11	W	Cubar^TUR^	Aerial parts	Infusion	Spasmolytic,	0	5	0	0	0.563	0	I
		Curbice^BOG^			Anti-diabetic,	0	2	0				
					Anti-parasitic,	0	2	0				
					Respiratory tract infections,	0	5	0				
					Anti-tussive,	0	2	0				
					Expectorant	0	2	0				
*Scrophularia nodosa* L*.* (Scrophulariaceae)	W	/	Aerial parts	Topically applied	“*Saraxha*” (cutaneous tuberculosis), Tuberculosis	0	3	2	0	0.094	0.043	I
*Sempervivum tectorum* L. (Crassulaceae) 71/Pz/2013	W	Bar veshi^ALB^	Leaves	Extracted with fat (cow or pig fat) - topically applied	Wound healing	6	3	0	0.279	0.531	0	I
		Cuvarkuca^BOG^										
				Juice from fresh leaves, 2–3 drops	Earache, ear infections	11	14	0				
*Symphytum officinale* L. (Boraginaceae) 73/Pz/2013	W	Crni gavez^BOG^ Ganez^TUR^	Roots	Extracted with fat	Wound healing	0	4	2	0	0.188	0.109	I
				Extracted with wine	Anticoagulant	0	2	3				
*Tanacetum vulgare* L*.* (Asteraceae) 75/Pz/2013	W	Pire otu^TURR^	Seeds	Infusion	Anti-parasitic (intestinal parasites),	0	0	3	0	0	0.304	I
					Anti-rheumatic	0	0	2				
			Flowers	Powder	Insect repellent, anti-parasitic	0	0	4				
			Aerial parts	Infusion	Digestive tract disorders,	0	0	2				
					Anti-hemorrhoidal,	0	0	1				
					Eczema	0	0	2				
*Taraxacum officinale* F.H. Wigg. (Asteraceae)84/Pz/2013	W	Tamëlçak i	Flowers	Infusion	Hepatitis	2	1	0	0.295	0.375	0.304	I
		livadhit^ALB^	Aerial parts	Infusion	Improve blood circulation,	7	4	9				
		Maslacak^BOG^										
		Karaındıba^TUR^			Digestive tract disorders,	3	5	1				
					Urinary tract disorders,	1	1	1				
					Anti-anemic.	5	1	3				
*Teucrium chamaedrys* L. (Lamiaceae) 79/Pz/2013	W	Mamudia^BOG^	Aerial parts	Infusion	Appetizing,	0	2	0	0	0.250	0	I
					Stomachache,	0	4	0				
					Anti- diarrheal,	0	1	0				
					Anti-hemorrhoidal	0	1	0				
*Teucrium polium* L. (Lamiaceae) 78/Pz/2013	W	Bar saraxha^ALB^	Aerial parts	Mixed with fat	Tuberculosis,	2	2	0	0.131	0.281	0	I
		Bar majasili^ALB^			“*Saraxha*” (cutaneous tuberculosis)	4	2	0				
		Podobica^BOG^										
				Infusion	Anti-hemorrhoidal,	1	1	0				
					igestive tract disorders,	1	3	0				
					Stomachache	0	1	0				
*Thymus serpyllum* L. (Lamiaceae) 76/Pz/2013	W	Majcina dusica^BOG^	Aerial parts	Infusion	Improve blood circulation,	3	5	2	1.525	2.5	1.087	I
		Qeklik oti^TUR^										
					Anticholesterolemic,	1	2	0				
					Respiratory inflammations,	21	26	16				
					Immunostimulant,	4	0	2				
					Neurorelaxant,	11	5	3				
					Carminative,	19	22	13				
					Spasmolytic,	13	9	12				
					Bronchitis,	16	6	1				
					Anti-asthmatic,	2	4	1				
					Expectorant	3	1	0				
*Thymus vulgaris* L. (Lamiaceae) 77/Pz/2013	W	Majcina dusica^BOG^	Aerial parts	Infusion	Anti-tussive,	0	3	0	0	0.281	0	I
					Anti-cholesterolemic	0	6	0				
*Typha latifolia* L. (Typhaceae) 82/Pz/2013	W	Shavar^ALB^	Fruits	Infusion	Respiratory system inflammations	0	0	3	0	0	0.065	I
		Hubabo^TUR^										
*Tilia platyphyllos* Scop. (Malvaceae) 80/Pz/2013	W/C	Blini^ALB^	Flowers	Infusion	Respiratory system inflammations,	8	13	8	0.689	1.469	0.804	I
		Lipa^BOG^										
		Flamur^TUR^			Anti-anemic,	2	5	4				
		Ilhamur^TUR^			Stomach infections,	9	3	5				
					Headache,	1	0	1				
					Anti-tussive	2	0	1				
			Leaves and Flowers	Infusion	Anti-tussive,	2	4	6				
					Expectorant,	1	3	1				
					Respiratory system inflammations	17	19	11				
*Trifolium arvense* L. (Fabaceae)	W/C	Tërfoja^ALB^	Aerial part	Infusion	Anti-rheumatic	0	6	0	0	0.188	0	I
		Deklina^BOG^										
*Triticum vulgare* L. (Poaceae) 74/Pz/2013	W	Gruni^ALB^	Flour	Mixed with hot water – topically used	Skin inflammation and ulcers	0	0	4	0	0	0.087	I
		Bogday^TUR^		Mixed with hot water – internal used	Anti-diarrheal	0	0	2				
*Tussilago farfara* L. (Asteraceae) 83/Pz/2013	W	Potbel^BOG^	Aerial parts	Infusion	Expectorant,	0	7	0	0	0.313	0	I
					Anti-tussive	0	3	0				
*Ulmus minor* Mill. (Ulmaceae) 86/Pz/2013	W	Vidhi^ALB^	Leaves	Extracted with fat	Anti-mycotic,	11	0	7	0.197	0	0.217	I
		Karragaq^TUR^			Anti-bacterial, “*Saraxha*” (cutaneous tuberculosis).	1	0	3				
*Urtica dioica* L. (Urticaceae) 85/Pz/2013	W	Hithi^ALB^	Aerial parts	Infusion	Anti-hemorrhoidal,	3	1	5	1.820	3.094	1.652	II
		Kopriva^BOG^			Anti-anemic,	32	21	11				
		Yakici^TUR^			Influenza,	12	10	6				
					Anti-cancer,	1	0	0				
					Eczemas,	3	7	9				
					Bronchitis,	11	19	14				
					Headache,	1	3	1				
					Anti-rheumatic,	9	4	6				
					Anti-bacterial,	12	19	13				
					Alopecia,	5	1	1				
					Anti-dandruff,	18	12	9				
					Digestive disorders	2	1	1				
					Urinary disorders	2	1	0				
*Vaccinium myrtillus* L. (Ericaceae) 87/Pz/2013	W	Boronıca^ALB^	Fruits	Juice of fresh fruits	Digestive tract infections,	6	9	5	0.984	1.563	1.152	I
		Borovnica^ALB^										
					Anti-anemic,	25	21	11				
					Eye inflammations,	1	0	5				
					Hepatitis,	0	0	3				
					Digestive disorders,	0	1	3				
					Urinary disorders	1	1	1				
			Fruits and leaves	Infusion	Lithontriptic,	4	2	7				
					Respiratory inflammations,	6	5	3				
					Anti-anemic	17	11	15				
*Vaccinium vitis-idaea* L. (Ericaceae)	W	Brusnica^BOG^	Leaves	Infusion	Urinary inflammations	0	14	0	0	2.094	0	I
					Anti-rheumatic	0	3	0				
			Fruits	Infusion	Urinary tract infections	0	21	0				
			Fruits and leaves	Infusion	Lithontriptic,	0	11	0				
					Diuretic,	0	6	0				
					Anti-rheumatic,	0	1	0				
					Wound healing,	0	3	0				
					Antipyretic,	0	1	0				
					Anti-diabetic,	0	6	0				
					Anticonvulsant	0	1	0				
*Veratrum album* L. (Melanthiaceae)	W	Shtara^ALB^	Aerial parts	Infusion	Anti-hypertensive.	3	5	0	0.049	0.156	0	I
		Cemenika^BOG^										
*Verbascum sp.* (Scrophulariaceae) 89/Pz/2013	W	Divizma^BOG^ Diviza^TUR^	Aerial parts	Infusion and Mixed with fat “mehlem”	Anti-tussive,	0	2	1	0	0.250	0.087	I
					Bronchitis,	0	5	2				
		Sigir kuyrugu^TUR^			Digestive tract disorders	0	1	1				
*Veronica officinalis* L. (Plantaginaceae) 88/Pz/2013	W	Paskalya otu^TUR^	Leaves	Infusion	Anticoagulant,	0	0	3	0	0	0.196	I
		Yavshan otu^TUR^			Respiratory system inflammations,	00	0	2				
					Wound healing		0	4				
*Vitis vinifera* L*.* (Vitaceae) 90/Pz/2013	C	Rrushı^ALB^	Leaves	Infusion	Increase immunity,	4	1	0	0.311	0.438	0.435	I
		Grozhgje^BOG^			Hepatitis	2	1	3				
		Siyah üzüm^TUR^										
			Fruits	Eaten fresh	Anti-anemic,	3	4	2				
					Hepatic disorders,	1	2	3				
					Urinary system inflammations	6	2	1				
			Juice of fruits (semi fermented)	Internal used	Anti-anemic,	1	1	10				
					Anti-cholesterolemic	2	3	1				
*Zea mays* L*.* (Poaceae) 92/Pz/2013	C	Misri^ALB^	Female flower	Infusion	Urinary tract inflammations,	2	0	3	0.115	0	0.152	I
		Kollomoq^ALB^										
					Edema,	1	0	1				
		Kollomoqi^TUR^			Stomach disorders,	2	0	0				
					Anti-parasitic	1	0	0				
			Ripe seeds	Infusion	Anti-parasitic	1	0	3				

^**a**^ **Folk Names.** ^ALB^folk name(s) recorded among Albanians; ^BOG^folk name(s) recorded among Bosniaks/Gorani; ^TUR^folk name(s) recorded among Turks

^**b**^ **Alb N**_**uc**_ **:** Number of use citations provided by Albanian informants; **Bo/Go N**_**uc**_ **:** Number of use citations provided by Bosnian and Gorani informants; **Tur N**_**uc**_ **:** Number of use citations provided by Turkish informants.

^**c**^ **UV**_**Alb**_ **:** Use-value for one species by the Albanian group; **UV**_**Bo/Go**_ **:** Use-value for one species by the Bosniaks and Gorani; **UV**_**Tur**_ **:** Use-value for one species by the Turkish group. This index measures the relative importance of each species based on its reported use by informants from each cultural group under study.

^**d**^ **Q:** Quadrant assignments are based on adjusted use-values (UV_adj_), which were calculated by dividing the use-value (UV) of each group by the maximum use-value (UV_max_) for medicinal citations (UV_adj_ not shown).

**Table 2:** **Wild plant or mushroom species used as local food in the study area**

**Botanical taxon, family and voucher specimen code**	**Folk name(s)** ^**a**^	**Part(s) used**	**Preparation**	**Folk uses(s)**	**Alb N**_**uc**_^**b**^	**Bo/Go N**_**uc**_^**b**^	**Tur N**_**uc**_^**b**^	**UV**_**Alb**_^**c**^	**UV**_**Bo/Go**_^**c**^	**UV**_**Tur**_^**c**^	**Q** ^**d**^
*Amanita caesarea* (Scop.) Pers. (Amanitaceae)	Kërpurdha^ALB^	Aerial parts	Fresh or conserved	Food used in small quantities,	3	0	0	0.08	0	0	I
				Food additive	2	0	0	2			
*Castanea sativa* Mill*.* (Fagaceae) 19/Pz/2013	Gështaja^ALB^	Fruits	Fresh, beaked	Food	6	4	4	0.098	0.125	0.087	I
*Cichorium intybus* L. (Asteraceae) 21/Pz/2013	Cikorja^SHQ^	Aerial parts	Dried and ground	Coffee substitute, prepared as Turkish coffee	2	0	3	0.033	0	0.065	I
	Mavi çiçek^TUR^										
	Satali bitki ^TUR^										
*Cornus mas* L. (Cornaceae) 23/Pz/2013	Thana^ALB^ Dirnina^TUR^	Fruits	Eaten fresh	Food	5	6	0	0.082	0.563	0	I
			Mixed and boiled with sugar for short period	Beverage	0	6	0				
			Mixed and boiled with sugar for longer period	Jam	0	6	0				
*Corylus avellana* L. (Betulaceae) 24/Pz/2013	Lejthı^ALB^	Fruits	Fresh or dried	Food, Sweetener	9	15	5	0.148	0.469	0.109	I
*Foeniculum vulgare* Mill*.* (Apiaceae) 32/Pz/2013	Kopër^ALB^ Rezene^BOG^	Leaves, seeds	Dried	Food additive for flavoring	0	0	2	0	0	0.043	I
	Anason^TUR^										
*Fragaria vesca* L. (Rosaceae)	Dreza^ALB^	Fruits	Eaten fresh	Food	15	19	7	0.295	1.406	0.196	II
	Divla jagoda^BOG^										
			Mixed and boiled with sugar for short period	Beverage	0	15	0				
			Mixed and boiled with sugar for longer period	Jam	3	11	2				
*Helianthus tuberosus* L*.* (Asteraceae)	Orashka^ALB^	Tuber	Eaten fresh	Food	3	0	0	0.033	0.25	0	I
*Malus sylvestris* Mill. (Rosaceae)		Fruits	Boiled with sugar	Jams	0	3	0	0.033	0.25	0	I
			Sliced and dried (*ahaf*), boiled in water prior to eating	Wintertime food	2	5	0				
*Matricaria chamomilla* L. (Asteraceae) 43/Pz/2013	Lule qeni^ALB^	Aerial parts	Dried	Tea	0	5	3	0	0.156	0.065	I
	Kamomil^ALB^										
	Papatja^TUR^										
	Sari çiçek^TUR^										
	Kamilica^BOG^										
	Babunec^BOG^										
*Mentha longifolia* (L.) Huds. (Lamiaceae) 45/Pz/2013	Çaj nana^ALB^ Nana^BOG^	Aerial parts	Infusion	Tea	0	0	3	0	0	0.065	I
*Morus alba* L. (Moraceae) 49/Pz/2013	Mani i bardhe^ALB^	Fruits	Fresh	Food	6	5	0	0.098	0.156	0	I
	Akdut^TUR^										
*Morus nigra* L*.* (Moraceae) 48/Pz/2013	Mani i zi^ALB^	Fruits	Eaten fresh	Food	8	9	4	0.131	0.406	0.109	I
	Dut^TUR^		Mixed and boiled with sugar for short period	Beverage	0	2	1				
	Karadut^TUR^										
			Mixed and boiled with sugar for longer period	Jam	0	2	0				
*Orchis morio* L. (Orchidaceae) 53/Pz/2013	Salepi^ALB^	Tubers	Dried	Hot beverage mixed with milk “*salep*”	0	6	11	0	0.188	0.239	I
*Origanum vulgare* L. (Lamiaceae) 52/Pz/2013	Çaj mali^ALB^	Aerial parts	Infusion	Tea, Food flavoring	8	13	0	0.131	0.406	0	I
	Toqilla^TUR^										
*Prunus spinosa* L. (Rosaceae)	Kulumrija^ALB^	Fruits	Eaten fresh	Food	4	8	0	0.066	0.25	0	I
	Ternina^BOG^										
	Kurumlia^TUR^										
*Pyrus communis* L. (Rosaceae) 58/Pz/2013	Dardha^ALB^	Fruits	Fresh, conserved	Food	6	12	6	0.098	0.375	0.130	I
	Dardha eger^ALB^										
*Rosa canina* L*.* (Rosaceae) 67/Pz/2013	Kaça^ALB^	Fruits	Infusion	Tea, Jam	0	6	4	0	0.188	0.087	I
	Shipak^BOG^										
	Sipurak^BOG^										
*Rosa damascena* Mill. (Rosaceae)	Trendafili^ALB^	Flowers	Flowers mixed with cold water for 24 hours, and then lemon juice is added	Juice (*shurup*)	5	0	0	0.082	0	0	I
*Rubus fruticosus* L. (Rosaceae) 65/Pz/2013	Mana^ALB^	Fruits	Eaten fresh	Food	6	5	2	0.098	0.281	0.043	I
	Kupina^BOG^										
			Mixed and boiled with sugar for short period	Beverage	0	4	0				
*Rubus idaeus* L. (Rosaceae) 66/Pz/2013	Mjedra^ALB^	Fruits	Eaten fresh	Food	5	4	4	0.082	0.219	0.109	I
	Malina^BOG^										
			Mixed and boiled with sugar for short period	Beverage	0	3	1				
*Sambucus nigra* L*.* (Adoxaceae) 69/Pz/2013	Shtogu^ALB^	Flowers	Flowers mixed with cold water for 24 hours, and then lemon juice is added	Beverage	9	11	8	0.148	0.344	0.174	I
	Zova^BOG^										
	BOG zova^BOG^										
	Murver^TUR^										
	Forboz^TUR^										
*Thymus serpyllum* L. (Lamiaceae) 76/Pz/2013	Qeklik oti^TUR^	Aerial parts	Infusion	Tea	0	13	0	0	0.406	0	I
	Majcina dushica^BOG^										
*Thymus vulgaris* L. (Lamiaceae) 77/Pz/2013	Majcina dushica^BOG^	Aerial parts	Infusion	Tea	0	8	0	0	0.25	0	I
*Tilia platyphyllos* Scop. (Malvaceae) 80/Pz/2013	Blini^ALB^	Flowers	Infusion	Tea	9	11	6	0.148	0.344	0.13	I
	Flamur^TUR^										
	Ilhamur^TUR^										
	Lipa^BOG^										
*Urtica dioica* L. (Urticaceae) 86/Pz/2013	Hithi^ALB^	Aerial parts	Young and fresh	Filling in home-made savory pies (*pite*)	14	11	11	0.23	0.344	0.239	I
	Kopriva^BOG^										
	Yakici^TUR^										
*Vaccinium myrtillus* L. (Ericaceae) 87/Pz/2013	Boronıca^ALB^ BorovnicaB	Fruits	Eaten fresh	Food	8	11	5	0.131	0.594	0.109	I
			Mixed and boiled with sugar for short period	Beverage	0	8	0				
*Vitis vinifera* L*.* (Vitaceae) 90/Pz/2013	Rrushı^SHQ^	Leaves	Fresh ore conserved	*Sarma* ingredient: leaves are rolled around a filling usually based on minced meat and rice.	9	12	10	0.148	0.375	0.217	I
	Grozhgje^BOG^										
*Zea mays* L*.* (Poaceae) 92/Pz/2013	Misri^ALB^	Flour	Semi-fermented	Beverage “*boza*”	0	6	8	0	0.188	0.174	I
	Kollomoq ^ALB^										
	Kollomoqi^TUR^										

^**a**^ **Folk Names.** ^ALB^folk name(s) recorded among Albanians; ^BOG^folk name(s) recorded among Bosniaks/Gorani; ^TUR^folk name(s) recorded among Turks.

^**b**^ **Alb N**_**uc**_ **:** Number of use citations provided by Albanian informants; **Bo/Go N**_**uc**_ **:** Number of use citations provided by Bosnian and Gorani informants; **Tur N**_**uc**_ **:** Number of use citations provided by Turkish informants.

^**c**^ **UV**_**Alb**_ **:** Use-value for one species by the Albanian group; **UV**_**Bo/Go**_ **:** Use-value for one species by the Bosniaks and Gorani; **UV**_**Tur**_ **:** Use-value for one species by the Turkish group. This index measures the relative importance of each species based on its reported use by informants from each cultural group under study.

^**d**^ **Q:** Quadrant assignments are based on adjusted use-values (UV_adj_), which were calculated by dividing the use-value (UV) of each group by the maximum use-value (UV_max_) for food citations (UV_adj_ not shown).

**Table 3:** **Plants used in handicraft applications in the study area**

**Botanical taxon, family and voucher specimen code**	**Status**	**Folk name(s)** ^**a**^	**Part(s) used**	**Use Category**	**Specific Use(s)**	**Alb N**_**uc**_^**b**^	**Bo/Go N**_**uc**_^**b**^	**Tur N**_**uc**_^**b**^	**UV**_**Alb**_^**c**^	**UV**_**Bo/Go**_^**c**^	**UV**_**Tur**_^**c**^	**Q** ^**d**^
*Abies alba* Mill. (Pinaceae) 14/Pz/2013	W	Bredhi^ALB^	Wood	Carpentry	Used for home construction and different home furniture	5	4	2	0.082	0.125	0.043	I
*Acer campestre* L. (Sapindaceae)	W	Panja^ALB^	Wood	Carpentry	Used for constructing musical instruments (“çifteli”, violin etc.)	2	0	0	0.033	0	0	I
*Alnus glutinosa* L. (Betulaceae)	W	Verri^ALB^	Twigs	Dye	Brown color used for textile coloring	2	1	1	0.033	0.031	0.022	I
*Beta vulgaris* L. (Amaranthaceae)	C	Rrepa^ALB^	Taproot	Dye	Red color, used for textile coloring	2	0	0	0.033	0	0	I
*Centaurea cyanus* L. (Asteraceae) 20/Pz/2013	W	Kokoçeli^ALB^	Flowers	Dye	Blue color, used for textile coloring	0	5	0	0	0.156	0	II
		Kicica^BOG^										
*Corylus avellana* L. (Betulaceae) 24/Pz/2013	W	Lejthı^ALB^	Stems	Handicraft	Used to construct baskets, usually large ones for carrying animal food	10	5	0	0.164	0.156	0	VI
*Cotinus coggygria* Scop. (Anacardiaceae) 64/Pz/2013	W	Dru boje^ALB^	Fruits	Dye	Yellow color, used for leather, wool and other textile coloring	2	0	3	0.033	0	0.065	I
		Ruj^TUR^										
		Boyaci sumak^TUR^										
*Juglans regia* L. (Juglandaceae) 40/Pz/2013	C	Arra^ALB^	Wood	Carpentry	Used for furniture preservation, this is characterized by a high aesthetic value.	3	2	2	0.082	0.063	0.043	I
			Fruit cortex	Dye	Coloring of hair, wool and cotton	2	0	0				
*Juniperus communis* L. (Cupressaceae) 39/Pz/2013	W	Gëllija^ALB^	Wood	Musical instrument	Used for construction of “*lahuta*”, a single-stringed musical instrument used in traditionally music.	2	0	0	0.033	0	0	I
*Lagenaria siceraria* (Molina) Standl. (Cucurbitaceae)	C	Pocerka^ALB^	Dried fruits	Liquid container	Fruits opened and used as a water container	6	8	4	0.098	0.25	0.087	II
*Morus alba* L. (Moraceae) 49/Pz/2013	C	Mani i bardhë^ALB^	Wood	Liquid container	Used to construct casks for storing alcohol, which gives it a characteristic light yellow color	4	0	1	0.066	0	0.022	I
		Akdut^TUR^										
*Morus nigra* L*.* (Moraceae) 48/Pz/2013	C	Mani i zi^ALB^ Dut^TUR^	Wood	Liquid container	Used to construct casks for storing alcohol, which gives it a characteristic light yellow color	4	0	1	0.066	0	0.022	I
		Karadut^TUR^										
*Pinus nigra* J.F. Arnold*.* (Pinaceae)	W/C	Pisha^ALB^	Wood	Carpentry	Used for home construction and construction of different furniture.	0	6	1	0	0.188	0.022	II
		Kara qam^TUR^										
*Polygonum aviculare* L. (Polygonaceae)	W	Madimak^BOG^	Aerial parts	Dye	Blue color, used for wool coloring	0	0	3	0	0	0.065	I
		Kusekmezi^TUR^										
*Pyrus communis* L. (Rosaceae) 58/Pz/2013	W	Dardha^ALB^	Wood	Musical instrument	Used for construction of “*Zurla*”, an oboe-like woodwind instrument.	2	0	0	0.033	0	0	I
		Dardha eger^ALB^										
*Rhamnus frangula* (Rhamnaceae)	E	Druni barutit^ALB^	Wood	Weaponry	Used as a gunpowder ingredient	0	0	1	0	0	0.022	I
		Barut agaqi^TUR^										
*Rubia tinctorum* L. (Rubiaceae)	W	Boj kuqe^ALB^ Crvenka^BOG^	Roots and fruits	Dye	Red color, used for textile coloring	0	4	0	0	0.125	0	I
*Salix purpurea* L. (Salicaceae)	W	Rakita^ALB^	Twigs	Handicraft	To construct different type of baskets	5	2	0	0.082	0.063	0	I
*Sambucus ebulus* L*.* (Adoxaceae)	W	Kinla^ALB^	Fruits	Dye	Blue color, used for textile coloring	0	4	0	0	0.125	0	I
		Crna zova^BOG^										
*Zea mays* L*.* (Poaceae) 92/Pz/2013	C	Misri^ALB^	Mature leaves	Handicraft	Used to construct different types of baskets	0	0	4	0	0	0.087	I
		Kollomoq ^ALB^										
		Kollomoqi^TUR^										

^**a**^ **Folk Names.** ^ALB^folk name(s) recorded among Albanians; ^BOG^folk name(s) recorded among Bosniaks/Gorani; ^TUR^folk name(s) recorded among Turks.

^**b**^ **Alb N**_**uc**_ **:** Number of use citations provided by Albanian informants; **Bo/Go N**_**uc**_ **:** Number of use citations provided by Bosnian and Gorani informants; **Tur N**_**uc**_ **:** Number of use citations provided by Turkish informants.

^**c**^ **UV**_**Alb**_ **:** Use-value for one species by the Albanian group; **UV**_**Bo/Go**_ **:** Use-value for one species by the Bosniaks and Gorani; **UV**_**Tur**_ **:** Use-value for one species by the Turkish group. This index measures the relative importance of each species based on its reported use by informants from each cultural group under study.

^**d**^ **Q:** Quadrant assignments are based on adjusted use-values (UV_adj_), which were calculated by dividing the use-value (UV) of each group by the maximum use-value (UV_max_) for handicraft citations (UV_adj_ not shown).

## Results and discussion

In total, TEK on the local uses of 124 taxa (belonging to 51 families) was recorded; of these, 114 species were used for medicinal purposes, 29 wild species for food, and 20 for handicrafts. Some of the cited species were used for multiple purposes. The total number of use citation (N_uc_) for each species is reported by ethnic group and category of use: medicinal (Table 1), food (Table 2), and handicraft (Table 3) applications.

### Medicinal plants

TEK on the recorded local uses of 114 medicinal plant taxa, representing 49 taxonomic families, are reported in Table 1. Of these species, *Achillea millefolium* L., *Sambucus nigra* L., *Urtica dioica* L., *Tilia platyphyllos* Scop., *Hypericum perforatum* L., *Matricaria chamomilla* L., *Thymus serpyllum* L.*,* and *Vaccinium myrtillus* L. were cited by more than 30% of the informants. Of the 114 cited for medicinal purposes, 44 are also included in the official Pharmacopoeia of Europe (European Pharmacopoeia. 6 ed.). The predominantly quoted botanical families were Rosaceae (13%), Asteraceae (11%), and Lamiaceae (10%). These same three “top” families were found to also be predominant among the wild medicinal taxa used in the folk medicine of the Albanian Alps (Kosovo), Alps in Montenegro, Albania, and in the Gollak region of Kosovo [13,17-19,25,26].

The total number species quoted by each ethnic group were roughly equivalent: 67, 66, and 71 for the Albanians, Turks and Bosniaks, respectively. Figure 3A illustrates the overlap in citation of medicinal plant among the three populations, with 10 species used only by Albanians, 18 by Turks and 21 only by Bosniaks/Gorani. Furthermore, common uses were shared between certain groups: 15 only between Albanians and Turks, 8 only between Bosniaks/Gorani and Turks and 17 only between Albanian and Bosniaks/Gorani. A total of 25 species were cited for medicinal use by all three study populations.

The most frequently cited medicinal uses referred to gastrointestinal (17.8%), respiratory (15.1%) ailments, heart disease (13.6%), illnesses affecting the urogenital system (12.4%) and the skin (10.5%). These categories were the most frequently quoted in the ethnobotanical studies conducted in Gollak (Kosovo) [26], while the gastrointestinal and respiratory troubles were also the most frequently quoted in the ethnobotanical studies conducted in the Albanian Alps (Kosovar, Montenegrin and Albanian sides) [13,17-19,25].

Our 3-D analysis of the data revealed that of the cited species, *Chamomila recutita* had the highest use-value across groups, and was assigned to Quadrant VI, demonstrating high value among Albanians and Bosniaks/Gorani, with moderately high (UV_adj_ = 0.46) use-value among the Turkish population studied as well. While most taxa fell into Quadrant I, representing low to moderate level use-values among all three populations, two additional species stood out from the majority and fell into Quadrant II: *Allium sativum* and *Urtica dioica.* Both of these taxa demonstrated high use-value scores among Bosniaks/Gorani , with moderate use-values among Albanians and Turks.

**Figure 5:**
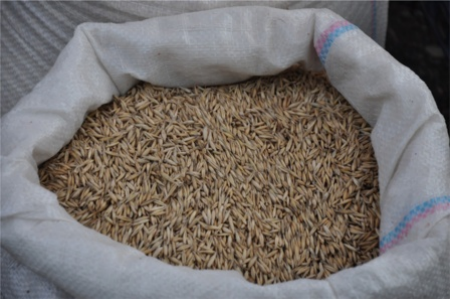
The aerial parts of *Avena sativa* L. (Poaceae) are prepared as an infusion and drunk for the purpose of enhancing the skeletal system.

**Figure 6:**
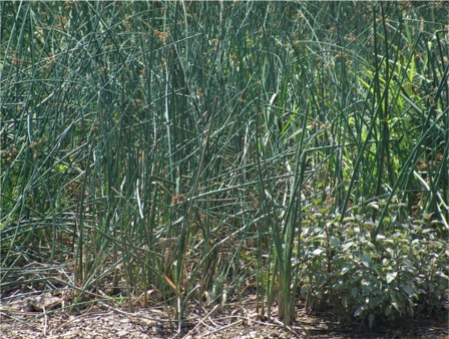
The aerial parts of *Juncus effusus* L. (Juncaceae) are prepared as an infusion and drunk to treat urinary tract disorders.

Upon cross-cultural comparative analysis of our findings with those reported in the medico-ethnobotanical literature available on the Southern Balkans [1-4,6,8-10,12,13,15,17,19-21,25-27], we identified the following novel uses of several plants, which could merit further phytochemical and bioactivity analyses:

- the topical application of the fruiting body of *Amanita caesarea* in the treatment of skin infections;
- the drinking of an infusion of the aerial parts of *Apium graveolens* to treat sterility;
- the drinking of an infusion of the aerial parts of *Avena sativa* (Figure 5)for its skeletal system enhancement effect;
- the consumption of *Brassica rapa* taproot to treat eye disorders and stimulate the immune system;
- the drinking of an infusion of aerial parts of *Geranium sanguineum to* treat respiratory disorders;
- the topical application of *Hordeum sativum* flour, mixed with oil, for wound healing;
- the drinking of an infusion of the aerial parts of *Juncus effusus* (Figure 6) to treat urinary tract disorders;
- the drinking of an infusion of the aerial parts of *Leonurus cardiaca* as cardiotonic, to improve blood circulation and memory enhancement; and
- the drinking of an infusion of aerial parts of *Trifolium arvense* as an anti-rheumatic.

### Food plants

The food uses of 29 wild species, representing 16 families, were recorded (Table 2). Of these, 3 were quoted only by Albanians, 2 only by Turks and 2 only by Bosniaks/Gorani. Figure 3B illustrates the high level of overlap of cited plant species for food uses, with 12 species being cited by all three populations. Regarding common overlaps in species uses, 1were shared in common only between Albanians and Turks, 4 only between Bosniaks/Gorani and Turks and 5 only between Albanian and Bosniaks/Gorani. Regarding the preparation of traditional foods, some of these, such as *salep* (beverage from *Orchis* spp. tubers) and *shurup* (syrup from *Rosa damascena* flowers), were prepared quite frequently in the past, but nowadays have nearly disappeared. The most frequently cited food uses of local plants referred to foods that are eaten fresh or processed (33.3%), beverages (22.2%), teas (17.8%), jams (17.8%) and food additives (8.9%). Our 3-D analysis of adjusted use values revealed that all taxa with the exception of one are placed in Quadrant I, indicating a common low to moderate level of use-value shared among populations. Wild strawberries (*Fragaria vesca*), on the other hand, fell in Quadrant II, and is highly valued by Bosniaks/Gorani for its use as a food, beverage ingredient and jam ingredient. Its use as a beverage by the Bosniak/Gorani, prepared by boiling with sugar, was not cited by either Albanians or Turks in this study.

### Handicraft plants

The handicraft uses of 20 species, representing 18 families, were recorded (Table 3). Of these, 5 were quoted only by Albanians, 3 by Turks and 2 by Bosniaks/Gorani. Figure 3C illustrates a moderate level of overlap of the handicraft uses of plant species, with only 4 being cited by all 3 populations. Regarding common overlaps in species uses, 3 were shared in common only between Albanians and Turks, 1 only between Bosniaks/Gorani and Turks and 2 only between Albanian and Bosniaks/Gorani. The most frequently cited form of handicraft uses of local flora included dyes (38.1%), musical instruments (28.6%), carpentry (19.0%) and liquid containers (14.3%).

As might be expected with lower levels of overlap between taxa cited for use for this purpose, we also observed greater distinction in the spread of taxa in our 3-D comparative analysis of adjusted use-values. Of note, *Corylus avellana,* which is a key resource for basket weaving in this region, fell into Quadrant VI, indicating its high use-value among Albanians and Bosniaks/Gorani. It had no cited use among Turks. *Lagenaria siceraria*, whose fruits are used as a container for carrying water, had a top use-value among Bosniaks/Gorani, with moderate scores among Albanians and Turks (Quadrant II). *Pinus nigra*, used for home and furniture construction, likewise has a high use-value score among Bosniaks/Gorani, but a very low use-value among Turks, and no citations for Albanians.

### Cross-cultural comparison

Both the distinct and overlapping patterns of TEK reported by the 3 ethnic groups are illustrated in Figure 3. Although the number of informants was slightly uneven among the three populations, a general tendency can be observed nevertheless, also because “saturation” plateaus in which no new plant uses quoted by new interviewees were commonly reached after approximately 15–20 interviews. While we could not observe any remarkable differences among the wild plants used in the food and handicraft domains by the three populations, a difference is notable in the medicinal domain. When it comes to medicinal TEK, Albanians appear less *herbophilic* than both Slavs and Turks. This finding confirms what has already been pointed out by other field studies conducted in other Western Balkans areas and involving both Slavs and Ghegh Albanians [10,15]. This phenomenon may be best explained by the fact that the traditional economy of Ghegh Albanians was for many centuries based upon a pure pastoralist/transhumant economy, whereas they have rarely traded herbs. For the Slavs, however, the gathering of herbs from the wild has persisted as their well-known main occupation within a mixed system of small-scale agriculture and pastoralism. This is especially the case among Islamicized Slavs living in the mountainous areas of SE Europe.

## Conclusion

For the first time in European ethnobotany, this study presents data comparing the medicinal, food, and handicraft plant use practices of three different ethnic populations living in the same area. We have introduced a new analytical method (3-D adjusted use-value plots) for comparison of taxa across different populations living in the same environment, with access to the same taxa and other environmental resources. While we have documented the presence of some small distinct sets of TEK in these populations, this is overwhelmingly coupled by a substantial overlap in the use of local taxa, suggesting a hybrid character to the Kosovar TEK in this region, especially with regards to TEK in the food and handicraft domains. Such cross-cultural studies could be important for proposing culturally-sensitive ways of using plant natural resources in future sustainable economic development initiatives. Indeed, the success of any future development efforts involving natural resources must take into account local perceptions and attitudes concerning plants, which can vary greatly in some cases, among different ethnic groups living in the same territory. Examples of such initiatives could include a focus on eco-tourism and the small-scale trade of foods, aromatic plants, medicinal herbs, and handicraft products. Findings from studies such as this one should be implemented in projects aimed at fostering collaboration and reconciliation among the diverse ethnic and religious communities living in Kosovo.
